# Second wave COVID-19 pandemics in Europe: a temporal playbook

**DOI:** 10.1038/s41598-020-72611-5

**Published:** 2020-09-23

**Authors:** Giacomo Cacciapaglia, Corentin Cot, Francesco Sannino

**Affiliations:** 1 Institut de Physique des 2 Infinis (IP2I), CNRS/IN2P3, UMR5822, 69622 Villeurbanne, France; 2 Université de Lyon, Université Claude Bernard Lyon 1, 69001 Lyon, France; 3 CP3-Origins & the Danish Institute for Advanced Study, University of Southern Denmark, Campusvej 55, 5230 Odense, Denmark; 4 Dipartimento di Fisica E. Pancini, Università di Napoli Federico II & INFN sezione di Napoli, Complesso Universitario di Monte S. Angelo Edificio 6, via Cintia, 80126 Naples, Italy

**Keywords:** Mathematics and computing, Physics

## Abstract

A second wave pandemic constitutes an imminent threat to society, with a potentially immense toll in terms of human lives and a devastating economic impact. We employ the *epidemic Renormalisation Group* (eRG) approach to pandemics, together with the first wave data for COVID-19, to efficiently simulate the dynamics of disease transmission and spreading across different European countries. The framework allows us to model, not only inter and extra European border control effects, but also the impact of social distancing for each country. We perform statistical analyses averaging on different level of human interaction across Europe and with the rest of the World. Our results are neatly summarised as an animation reporting the time evolution of the first and second waves of the European COVID-19 pandemic. Our temporal playbook of the second wave pandemic can be used by governments, financial markets, the industries and individual citizens, to efficiently time, prepare and implement local and global measures.

## Introduction

A second wave pandemic constitutes an imminent threat to society, with an immense toll in terms of human lives and a devastating economic impact. The disease diffusion dynamics is traditionally modelled via compartmental^1^ or complex network diffusion techniques^2–4^. These models provide a fairly accurate description of the time evolution of the number of affected individuals. However, it is a hurdle to predict the future evolution of a pandemic^5^ and to account for the diffusion across different regions of the World. Here we show that the epidemic Renormalisation Group framework^6,7^ is a simple and effective method to provide robust projections of the time evolution of a pandemic across regions. We apply it to the COVID-19, calibrating it on the first wave data, to efficiently simulate an incumbent second wave across Europe. We perform statistical analyses averaging on different levels of human interaction across Europe and with the rest of the world, finding that the second wave will occur between July 2020 and January 2021. Our results demonstrate that our method can be employed to describe pandemic dynamics beyond the European example. We anticipate that our results can be functional to a more quantitative understanding of future pandemics, which are expected to become a recurrent threat to our society. Our temporal playbook of the second wave pandemic can be used by governments, financial markets, the industries and individual citizens, to efficiently time, prepare and implement local and global measures.

Pandemics are increasingly becoming a constant menace to the human race, with COVID-19^8,9^ being the latest example. A second wave is creeping back in Europe and is poised to rage across the continent by fall 2020. In this letter we provide a statistical analysis of the temporal evolution of the second wave of infected cases, with the impact for various European countries. To model the spreading, we employ the *epidemic Renormalisation Group* (eRG) framework^6,7^. It can be mapped^7,10^ into a time-dependent compartmental model of the SIR type^1^. The Renormalisation Group approach^11,12^ has a long history in physics with impact from particle to condensed matter physics and beyond. Its application to epidemic dynamics is complementary to other approaches^2–4,13–20^.

The eRG approach consists in a set of first order differential equations apt to describe the time-evolution of the infected cases in a specific isolated region. It has been extended^7^ to include interactions among multiple regions of the World, without the need for powerful numerical simulations. The set of equations^7^ reads

1 $$\begin{aligned} \frac{d \alpha _i}{d t} = \gamma _i \alpha _i \left( 1-\frac{\alpha _i}{a_i} \right) + \sum _{j\ne i} \frac{k_{ij}}{n_{mi}} (e^{\alpha _j - \alpha _i} -1) \quad \text{ where } \quad \alpha _i(t) = \mathrm ln\ {{\mathscr {I}}}_i(t) \ , \end{aligned}$$

with ${{\mathscr {I}}}_i (t)$ being the total number of infected cases *per million* inhabitants for region *i* and $\ln$ indicating its natural logarithm. These equations embody, within a small number of parameters, the pandemic spreading dynamics across coupled regions of the World via the temporal evolution of $\alpha _i$, which resembles the energy dependence of the couplings appearing in fundamental interactions of particle physics.

The first term of the right-hand side in Eq.(1) characterises the epidemic evolution within a given region of the World. The infection rate $\gamma _i$, measured in inverse weeks, is responsible for how quickly the epidemic evolves in the *i*-th region. Besides depending on the intrinsic virulent character of the epidemic, the size of $\gamma _i$ can be controlled via social-distancing measures, with a flatter epidemic curve associated to smaller $\gamma _i$. It is well understood^1^ that epidemic diffusion curves generally lead to plateaus in the total number of infected cases at late times. This is encoded in the parameter $a_i$, equal to the natural logarithm (ln) of the total number of infected cases (per million) at the end of the epidemic wave.

**Figure 1:**
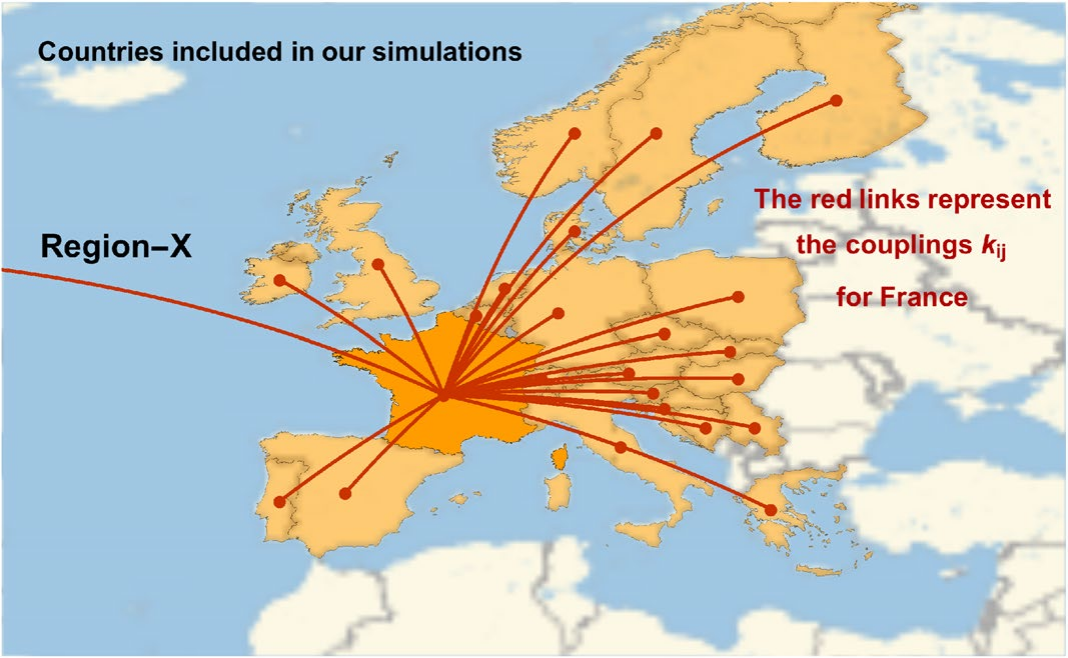
Illustration of the connections $k_{ij}$ between, i.e., France and the other countries considered in this study. Each line represents the exchange of infected cases. The line pointing outside the map represents the connection with Region-X, representing an inflow from a source outside the pool of countries in the simulation and/or the presence of hotspots in the concerned country. Figure created with Wolfram Mathematica.

The second term of the right-hand side in Eq.(1), first introduced here^7^, is a source-term that takes into account human interaction across different regions of the World. Here, $n_{mi}$ is the population of region-*i* in millions and $k_{ij}$ represents the number of reciprocal travellers per week from region *i* to region *j* and vice-versa in units of million people. For a single country, i.e. France, we illustrate diagrammatically the connections given by the $k_{ij}$ couplings in Fig. 1. We also consider an extra-source of infection modelled as a new region that we call Region-X ($i=0$). We can interpret this region in various ways: for instance, this may represent an inflow of infections coming from outside of the regions of the World included in the simulation or, alternatively, Region-X may represent the effect of local hotspots of infections. Of course, it could also be a combination of the two effects.

To simulate the second wave diffusion in Europe, we take, as reference, the values of the parameters $\gamma _i$ and $a_i$ to the first wave fits, which provides a reasonable benchmark for quantities that are very hard to predict^5^. The couplings $k_{ij}$, are randomly generated within the range $10^{-3}$–$10^{-2}$, which has been shown^7^ to give reasonable timing for the peak diffusion. We will consider five scenarios, which differ for the values given to the couplings to Region-X: (a) we use the randomly generated $k_{0i} = k_{i0}$, in the range $10^{-2} - 10^{-3}$; (b) we divide the $k_{0i}$ by a factor of ten, implying a 90% reduction of the interaction with Region-X; (c) we divide the $k_{0i}$ by a factor of hundred, i.e. a 99% reduction; (d) for unrestricted $k_{i0}$, we allow the $\gamma _i$’s to vary within $14\%$ of the first wave fit value; (e) all the $k_{0i}$ are set to zero except for a set of 10 countries, which already show signs of a second wave as of the 5th of August, 2020. For these countries, which are Belgium, Bosnia, Croatia, Czechia, Greece, the Netherlands, Serbia, Slovakia, Slovenia and Spain, the $k_{i0}$ and other parameters are tuned in order to fit the available data from the second wave, as detailed in the section “Methods”. We consider this case (e) as the most realistic one.

**Table 1:** Left block: parameters fitted from the first wave. Right block: median peak time of the second wave for the 5 typologies (cases a–e) we use in the simulations, with 1 standard deviation. For cases (a–c), the median and error only take into account the 100 simulations, differing by randomly generated matrices $k_{ij}$. For case (d), we include a variation of $15\%$ in the $\gamma _i$’s for all countries with respect to case (a). For case (e), we include the interval spanned by varying the $\gamma _i$’s within $10\%$ from the fitted values, where the results marked with an asterisk correspond to the tuned countries with a beginning of second wave, as of the 5th of August, 2020.

First wave parameters			Second wave simulations: peak timing (calendar weeks 2020)				
	*a*	$\gamma$	Case a	Case b	Case c	Case d	Case e
Austria	$7.463\pm 0.007$	$0.99\pm 0.025$	$30.4\pm 0.4$	$32.4\pm 0.5$	$34.7\pm 0.5$	$30.6\pm 0.9$	$34.8+0.9-0.7$
Belgium	$8.53\pm 0.02$	$0.55\pm 0.02$	$34.6\pm 0.7$	$37.9\pm 0.6$	$41.4\pm 0.5$	$34.9\pm 1.4$	$33.8^*+0.8-0.7$
Bosnia	$7.88\pm 0.024$	$0.41\pm 0.02$	$33.9\pm 0.6$	$37.4\pm 0.5$	$41.0\pm 0.5$	$34.0\pm 1.1$	$33.4^*+0.7-0.6$
Croatia	$6.268\pm 0.007$	$0.71\pm 0.02$	$30.9\pm 0.6$	$33.5\pm 0.7$	$36.6\pm 0.7$	$31.1\pm 1.0$	$27.7^*+0.1-0.1$
Czechia	$9.085\pm 0.014$	$0.56\pm 0.03$	$33.5\pm 0.7$	$36.8\pm 0.6$	$40.2\pm 0.5$	$33.7\pm 1.2$	$32.7^*+0.6-0.5$
Denmark	$7.667\pm 0.008$	$0.40\pm 0.01$	$35.6\pm 0.6$	$39.2\pm 0.5$	$42.7\pm 0.5$	$35.8\pm 1.2$	$39.9+1.3-1.1$
Finland	$7.190\pm 0.005$	$0.385\pm 0.006$	$35.4\pm 0.6$	$39.0\pm 0.5$	$42.5\pm 0.5$	$35.7\pm 1.2$	$39.8+1.3-1.1$
France	$7.711\pm 0.006$	$0.58\pm 0.012$	$36.0\pm 0.6$	$39.3\pm 0.5$	$42.6\pm 0.4$	$36.3\pm 1.4$	$41.1+1.4-1.2$
Germany	$7.679\pm 0.007$	$0.62\pm 0.02$	$35.8\pm 0.6$	$39.0\pm 0.5$	$42.3\pm 0.5$	$36.1\pm 1.5$	$39.9+1.4-1.1$
Greece	$5.537\pm 0.009$	$0.57\pm 0.02$	$32.5\pm 0.6$	$35.8\pm 0.5$	$39.2\pm 0.4$	$32.7\pm 1.0$	$31.7^*+0.6-0.5$
Hungary	$6.022\pm 0.009$	$0.47\pm 0.01$	$33.9\pm 0.6$	$37.4\pm 0.5$	$40.9\pm 0.5$	$34.2\pm 1.0$	$38.1+1.1-0.9$
Ireland	$8.580\pm 0.008$	$0.60\pm 0.02$	$33.1\pm 0.7$	$36.1\pm 0.7$	$39.5\pm 0.6$	$33.4\pm 1.2$	$36.6+1.0-0.9$
Italy	$8.304\pm 0.004$	$0.429\pm 0.008$	$39.1\pm 0.6$	$42.8\pm 0.4$	$46.1\pm 0.5$	$39.5\pm 1.7$	$43.9+1.7-1.4$
Netherlands	$7.904\pm 0.005$	$0.525\pm 0.008$	$35.2\pm 0.7$	$38.6\pm 0.6$	$42.1\pm 0.5$	$35.5\pm 1.3$	$34.3^*+0.9-0.7$
Norway	$7.356\pm 0.006$	$0.58\pm 0.02$	$32.7\pm 0.6$	$35.8\pm 0.7$	$39.3\pm 0.6$	$32.9\pm 1.0$	$36.4+1.0-0.8$
Poland	$7.13\pm 0.03$	$0.182\pm 0.007$	$45.7\pm 0.5$	$49.3\pm 0.6$	$52.6\pm 0.6$	$46.2\pm 1.7$	$54.6+2.7-2.2$
Portugal	$10.323\pm 0.014$	$0.517\pm 0.022$	$34.7\pm 0.7$	$38.1\pm 0.6$	$41.6\pm 0.5$	$35.0\pm 1.4$	$38.7+1.2-1.0$
Serbia	$9.323\pm 0.012$	$0.628\pm 0.017$	$32.6\pm 0.6$	$35.6\pm 0.6$	$38.9\pm 0.5$	$34.0\pm 1.1$	$29.2^*+0.3-0.3$
Slovakia	$5.67\pm 0.02$	$0.59\pm 0.04$	$31.7\pm 0.7$	$34.8\pm 0.7$	$38.2\pm 0.6$	$31.9\pm 1.0$	$30.7^*+0.5-0.4$
Slovenia	$7.299\pm 0.007$	$0.656\pm 0.017$	$30.7\pm 0.6$	$33.5\pm 0.7$	$36.7\pm 0.6$	$30.9\pm 0.9$	$29.7^*+0.3-0.3$
Spain	$8.747\pm 0.008$	$0.46\pm 0.01$	$38.2\pm 0.7$	$41.8\pm 0.5$	$45.2\pm 0.5$	$38.5\pm 1.8$	$33.8^*+0.8-0.7$
Sweden	$11.56\pm 0.04$	$0.162\pm 0.008$	$47.8\pm 0.5$	$51.3\pm 0.5$	$54.6\pm 0.6$	$48.3\pm 2.2$	$55.8+2.9-2.4$
Switzerland	$8.196\pm 0.003$	$0.72\pm 0.01$	$32.4\pm 0.6$	$35.1\pm 0.7$	$38.1\pm 0.7$	$32.6\pm 1.2$	$35.8+1.0-0.8$
UK	$8.353\pm 0.007$	$0.368\pm 0.007$	$41.0\pm 0.6$	$44.6\pm 0.4$	$48.0\pm 0.5$	$41.3\pm 1.9$	$46.2+2.0-1.6$

In all cases, the simulation starts in week 25, where no country is yet in the second wave. For the first four cases, we average over the 100 simulated matrices $k_{ij}$ to extract the location of the peak of the newly infected cases for the second wave per each country and the relative error. In case (d), we also include the error coming from the variation of $\gamma _i$. Finally, for case (e), the error comes from a $10\%$ variation for $\gamma$’s of all countries. The results are summarised in the last five columns of Table 1 with the errors representing one standard deviation. The time is given in 2020 calendar weeks.

## Results

**Figure 2:**
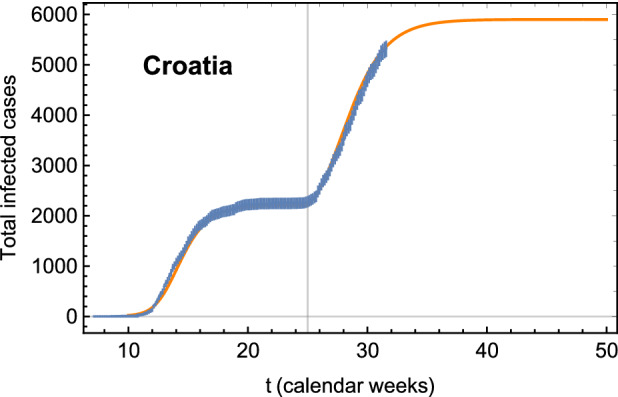
Croatian number of total infected cases (updated to the 5th of August) with respect to the theoretical curve (orange line) used to calibrate the case (e) simulation. The number of cases refer to the total population of Croatia. The vertical line shows where the second wave simulation begins.

We first discuss the results for the simulations in case (e), which are more realistic *vis à vis* the current situation in Europe, as of week 32 (i.e., the 5th of August). As already mentioned, we tuned the $k_{i0}$ and other parameters ($\gamma _i$ and $a_i$) for the second wave simulation in order to reproduce the data available between week 25 (the start of the simulation) and week 32. This was done to render the simulation more realistic. As an example, in Fig. 2 we show the outcome for Croatia compared to the actual data points (from www.worldometers.info). We also include the first wave from the fit. To obtain this result, we fixed $k_{i0} = 0.1$, and for the second wave we rescaled $\gamma$ and *a* respectively by 0.6 and 1.06 for Croatia alone. This implies that Croatia has already internal hotspots (as indicated by the large value of the coupling $k_{i0}$ with Region-X) and the second wave shows a smaller infection rate. For Croatia we also observe, however, that the total number of infected cases for the second wave is higher than for the first wave. It would be interesting to learn, from future data, whether this worrisome trend is followed by other European countries. The figure demonstrates that the result of our simple simulation can be tuned to reproduce the beginning of the second wave already observed in some countries. We have repeated the same tuning for Belgium, Bosnia, Czechia, Greece, the Netherlands, Serbia, Slovakia, Slovenia and Spain.

**Figure 3:**
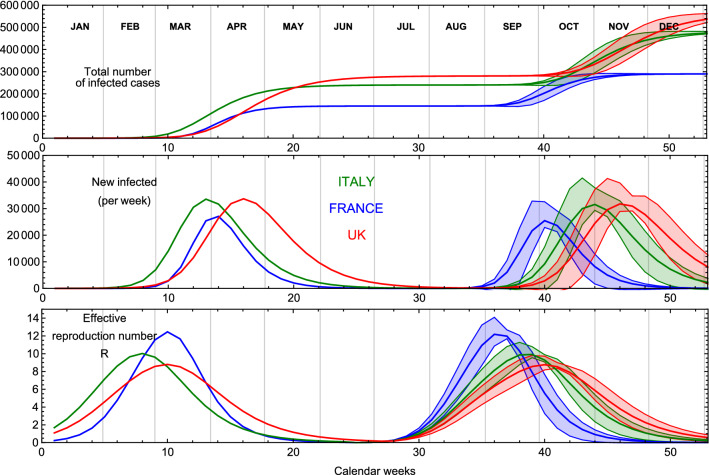
Result of case (e) for France, Italy and the UK. We show the time evolution of the total number of infected cases in the top panel, the new infected in the central panel and the derived effective reproduction rate *R* in the bottom panel. The number of cases refer to the total population of the countries. The bands are generated by varying the infection rates $\gamma _i$ within $10\%$.

As an example of our prognoses for the remaining countries, we show in Fig. 3 the epidemic dynamics of the first and second wave for three representatives: Italy, France and the UK. The top panel shows the number of infected cases not normalised per million. The central panel shows the number of new infected cases while the lower panel displays an estimate for the effective reproduction rate *R*. The number of recovered cases ${{\mathscr {R}}}(t)$ is calculated by solving the following SIR-inspired equation^7^:

2 $$\begin{aligned} \frac{d {{\mathscr {R}}}}{d t} = \epsilon \left( e^{\alpha (t)} - {{\mathscr {R}}}(t) \right) \,, \end{aligned}$$

where we fix the recovery rate $\epsilon =0.1$ in the numerical solutions. The effective reproduction rate *R* is estimated by computing the ratio of the new infected cases over the new recoveries within the susceptible population, from the theoretical model. The susceptible population is here defined as the total number of people infected at late time for the first and second waves independently. A more accurate result could be obtained using a generalised eRG approach^10^, at the expense of introducing more parameters. The plots are obtained using the simulations for case (e). Note that we used the same $\gamma$’s and *a*’s stemming from the first wave fit with the main error on the curve deriving from allowing a $10\%$ variation for the infection rates $\gamma$’s. One can also modify the values of *a*’s which will not modify the general temporal picture and trend of the second wave pandemic.

**Figure 4:**
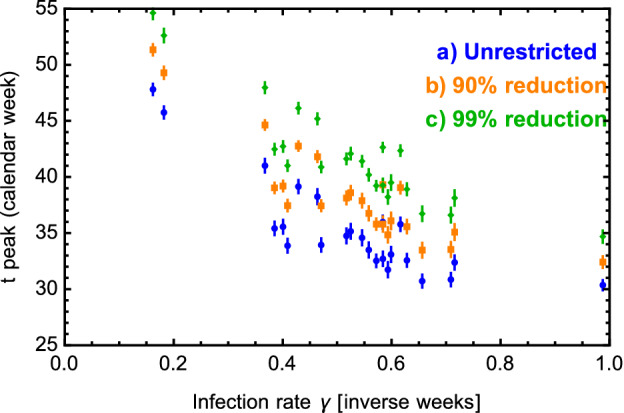
Peak time, in calendar weeks, versus the infection rate $\gamma$ for cases (a–c). These results are obtained by averaging the outcome of the 100 simulation, while the error bars indicate one standard deviation.

To study the dependence of the peak timing on $k_{ij}$, $\gamma _i$ and $a_i$, we can use the results from cases (a–c) from Table 1, as visualised in Fig. 4. Here we show the average peak time in calendar weeks versus $\gamma$ for all the countries in this study. Comparing the results in each set of simulations, we discover a clear correlation between the timing of the peak and the infection rate $\gamma _i$ of each country. The higher is the infection rate the sooner the peak is reached, as expected^6^. Furthermore, comparing the results for the three cases, we show that reducing the coupling with Region-X systematically delays the peaks, in accordance with previous results^7^. Quantitatively a reduction of a factor ten in the coupling to Region-X delays the peaks by about three weeks. We recall that, following the possible interpretations of Region-X, a reduction of the couplings to this region can be seen as the effect of travel bans and/or better control of local hotspots. Overall the peak timing ranges from end of July 2020 to beginning 2021. We did not find any correlation between the peak timing and the value of $a_i$ across the countries we studied. We also notice that the second wave peak timing for the countries without early signs of a second wave in case (e), indicated by un-starred values in Table 1, is close to the result found for case (c). This can be explained by the values of the $k_{ij}$ we use in case (e) simulation, which are close to the $k_{i0}$ used in case (c). The countries with early signs of a second wave act as a effective “Region-X” for the others.

The results from the first three simulations show a fairly small error in the peak time prediction due to the uncertainty on our knowledge of the $k_{ij}$ couplings. We recall that we vary the couplings within a factor of 10. However, the peak position also depends crucially on the value of the infection rates $\gamma _i$^6^. In this study, we use as a benchmark the values obtained by fitting the first wave. However, such values can depend crucially on the social distancing measures imposed in each country, so important variations are expected. For instance, it was shown in the eRG framework that reduction of individual mobility may lead to a decrease in $\gamma$ by $20\%$ or more^21^. For analogous analyses within compartmental models, see^22,23^. To study the robustness of our results with respect to a change in $\gamma$, we performed the simulation in case (d), where the $k_{ij}$ are as for case (a) while we allow the $\gamma _i$ to vary around the first wave value by $15\%$, following a Normal distribution. The results are shown in the second-to-last column of Table 1: while the central values agree with case (a), as expected, the error is substantially increased. This results proves that it is the value of $\gamma$ that can mostly influence the position of the second wave peak, thus highlighting the importance of timely social distancing measures. For the realistic simulation of case (e), we include a variation of $\gamma _i$’s by 10%, resulting in the intervals reported in the last column of Table 1. This can be considered our prognosis for the second wave in Europe. It should be clear that the *a*’s and the $\gamma$’s chosen for the simulation can, and will, be different from the first wave values we used. Nevertheless, we expect the dynamics to be still well represented by the framework and that these values give a reasonable indication for the second wave European pandemic.

## Discussion and video simulation

We employed the epidemic Renormalisation Group approach to simulate the dynamics of disease transmission and spreading across different European countries for the second COVID-19 wave. Since it has been demonstrated^10^ that the framework can be mapped into other compartmental models, our results are sufficiently general. The approach allows to model inter and extra European border control effects while taking into account the impact of social distancing for each country. To reduce the number of unknowns in the simulation, we used the information from the first wave. This information is encoded in the infection rate and the logarithm of the number of total infected cases per each country. Going beyond this hypothesis is straightforward in our approach, but such parameter tuning is not the point of this work. Nevertheless we allowed variations of the central value of $\gamma$’s by up to 15% to estimate the impact on the second wave pandemic and shown that the general overall trend remains unchanged. We then performed statistical analyses averaging on different level of cross Europe interactions and with the rest of the World. The role of the rest of the World and possibly local hotspots has been attributed to a Region-X, which acts as a source of infection coupled to all or only few European countries. By calibrating on the current European situation that shows early signs of the second wave, we provided a temporal playbook of the second wave pandemic. Our results can be employed by governments, financial markets and the industry world to implement local and global measures.

**Figure 5:**
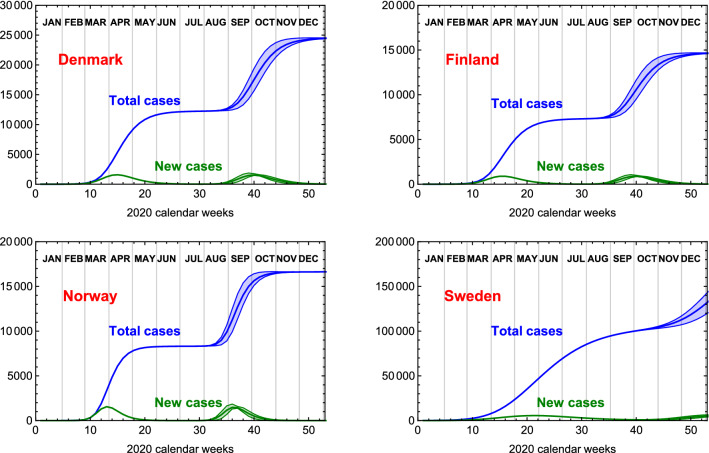
Time evolution for the total number of infected and new cases (not normalised per million) for the nordic European countries, from the simulation case (e), which starts at week 25. The bands are generated by varying the infection rates $\gamma _i$ within $10\%$.

The main results show that the temporal position of the second wave peak, once started, is rather solid and will occur between July 2020 and January 2021. As an example, we show in Fig. 5 our prognosis for the nordic countries, Denmark, Finland, Norway and Sweden. The precise timing for each country can be controlled via travel and social distancing measures. The sensitivity of the second peak prognosis on the value of the infection rates gives a clear indication that social distancing measures and responsible individual behaviour can have a strong effect if implemented early on. The predictions given by our model can, therefore, be easily updated to take into account the current situation in each country.

In the added material, we also include two animations representing the time evolution of the first and second wave of the European COVID-19 pandemic resulting from our simulations, extracted from case (a) and case (e) respectively, tuned to give the most realistic results and taking into account early signs of a second wave in some countries, as of the 5th of August, 2020. The simplicity of the eRG approach is such that the simulations take only a few seconds on an average personal laptop, thus providing a practical and accurate tool for the understanding of a second (and third, and so on) wave pandemic. The temporal playbook we provide is a useful tool for governments, financial markets, the industries and individual citizens to prepare in advance and possibly counter the threat of recurring pandemic waves.

## Note added

**Figure 6:**
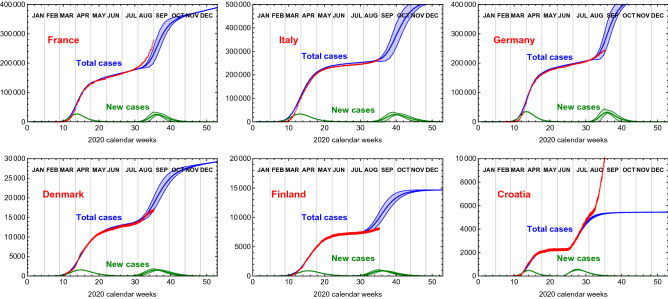
Epidemiological data (red), adjourned to the 30th of August, for six sample countries compared to an updated simulation. For all countries, except Croatia, the second wave from case (e) simulation is anticipated by 4 weeks, thus in agreement with the results of case (a). To match the slow growing phase in between the two waves, we added a linearly growing term, in line to what we did for the calibration of the case (e) simulations. The bands are generated by varying the infection rates $\gamma _i$ within $10\%$.

At the time of publication, many of the countries we considered in this study did enter a second wave of COVID-19 infections. By comparing the current data to our simulation case (e), we found that the second wave has started roughly 4 weeks earlier, compatible with case (a), for the countries that did not have signs of a second wave as of the 5th of August. Case (a) corresponds to values of the couplings $k_{ij}$ an order of magnitude larger than those used in case (e). One can see from Table 1 that the peak timing expected in case (a) reproduces better the observed data. To better appreciate this fact, in Fig. 6 we show the epidemiological data for six countries, adjourned to the 30th of August, compared to the simulation.

## Methods

To simulate the European second wave, we use as input parameters the values of $\gamma _i$ and $a_i$ stemming from the first wave. Predicting these parameters for the second wave is hard, as shown, for instance, via a *stochastic SEIR* model where very large fluctuations are found^5^. This is one of the reasons why we choose for our simulations the parameters coming from the first wave. Additionally this choice has the advantage of endowing us with reasonable benchmark values. These parameters depend on social distancing measures enacted by each country during the first wave. The methodology of the fit for $\gamma _i$ and $a_i$ is described in^6,7^. The values are reported in the first three columns of Table 1 at 90% confidence level. For the simulations we used the central values. The countries selected for this study, as listed in Table 1, are the ones whose data gives a good fit on the first wave and have a population above 3 millions to improve on the statistics. Some countries included in the study, namely Belgium, Bosnia, Croatia, Czechia, Greece, the Netherlands, Serbia, Slovakia, Slovenia and Spain, already show signs of a second wave starting between week 25 and week 32 (the 5th of August). Fur these countries we limit the data in the fit to the first wave. For the same reason, we started the simulation at week 25.

We now move to the interaction across the different European countries encoded in the matrix $k_{ij}$. We generate the entries of the matrix randomly with each value in the interval $10^{-3} - 10^{-2}$ and a flat probability. This translates in a range of 1k to 10k travellers per week across countries. In our earlier work^7^ this interval was shown to be able to account for the peak delay in between countries. As mentioned earlier, we also consider the extra-source of infection Region-X ($i=0$) with a fixed number of infected cases. This region couples to the different European countries with randomly generated $k_{0i} = k_{i0}$ in the same range as above. To Region-X we can assign different interpretations. One could be that of an extra-European source (say the rest of the World) that still couples to some or all European countries we consider. Another interpretation is that the coupling $k_{0i}$ to Region-X represents an internal source of infection inside the *i*-th region. To provide a sensible value for the initial source, we considered the current number of total infected (5.2 millions) normalised to the world population in millions.

Specifically, for the simulations cases (a–c), we randomly generate 100 copies of the matrix $k_{ij}$ to be used to repeat the simulation. The initial time of the second wave simulations is the calendar week 25, where we set the initial values for $\alpha _i = 0$ (while $\alpha _0 = \mathrm{constant}$). The 100 simulations are repeated, with the same set of $k_{ij}$ matrices for the first four cases described in the main text.

For case (d), we additionally include a variation in the values of the $\gamma _i$’s for the European countries. To do so, we generate randomly 100 sets of scaling factors $\epsilon _i$, one per country. The values of $\epsilon _i$ are generated randomly following a Normal distribution with mean 1 and standard deviation $\sigma = 0.15$. This allows for an uncertainty of $15\%$ on the value of the second wave $\gamma$ for each country.

For the realistic simulation, case (e), our strategy is a bit different. We fix $k_{ij} = 0.5 \times 10^{-3}$ for the couplings intra European countries. Furthermore, all $k_{i0} = k_{0i} = 0$, except for the 10 countries that already show a second wave start. For these countries we find the values of $k_{i0}$ and rescale the $\gamma$ and *a* parameters to fit the data adjourned to the 5th of August, 2020. One example of the fit is show in Fig. 2 for Croatia. Many countries, after reaching the peak of the first wave, feature a period with a linear growth of the infected cases. To better fit the second wave data, we included such a period, by adding the following term to the solution at the time when the first wave reaches the plateau:

3 $$\begin{aligned} \delta {{\mathscr {I}}} ( t>t_{\mathrm{pl}} ) = \theta _i (t-t_{\mathrm{pl}})\,. \end{aligned}$$

The values of the $k_{i0}$, rescaling of $\gamma$, *a*, and the parameters of the linear growth for the 10 countries in this study are as follows:

**Table:** 

	Belgium	Bosnia	Croatia	Czechia	Greece	Netherland	Serbia	Slovakia	Slovenia	Spain
$k_{i0}$	0.01	0.12	0.3	0.1	0.01	0.01	0.5	0.02	0.035	0.3
$\gamma _i$ scaling	1	0.7	0.6	0.6	1	1	0.7	0.8	0.6	0.85
$a_i$ scaling	1	1.3	1.06	1.06	1	1	1.05	1	0.95	1
$\theta _i$	60	0	0	40	7	35	35	1	1	30
$t_{\mathrm{pl}}$	20	−	−	20	20	20	20	20	20	20

Plots comparing our theory curve with the data are provided in the additional material.

## Supplementary information

Supplementary file1

Supplementary file2

Supplementary file3

## Data Availability

For the simulations, we used a code written for Wolfram Mathematica. The data of the simulations and a Mathematica code for their analysis can be made available upon request. The data for the COVID-19 infected cases in Europe are extracted from the worldometer.info repository.
