# Supplementary material for: Second wave COVID-19 pandemics in Europe: a temporal playbook
Source: Sci Rep. 2020 Sep 23;10:15514. doi: 10.1038/s41598-020-72611-5 (PMC7511360; doi:10.1038/s41598-020-72611-5)
Supplement: Supplementary file 1 — Supplementary file1 [file 41598_2020_72611_MOESM1_ESM.pdf]

Second wave COVID-19 pandemics in Europe: A Temporal Playbook

Giacomo Cacciapaglia[1,2,*,+], **Corentin Cot**[1,2,+], **and Francesco Sannino**[3,4,+]

[1]Institut de Physique des 2 Infinis (IP2I), CNRS/IN2P3, UMR5822, 69622 Villeurbanne, France
[2]Université de Lyon, Université Claude Bernard Lyon 1, 69001 Lyon, France
[3]CP3-Origins & the Danish Institute for Advanced Study, University of Southern Denmark, Campusvej 55, DK-5230 Odense, Denmark
[4]Dipartimento di Fisica E. Pancini, Università di Napoli Federico II & INFN sezione di Napoli, Complesso Universitario di Monte S. Angelo Edificio 6, via Cintia, 80126 Napoli, Italy
[*]g.cacciapaglia@ipnl.in2p3.fr
[+]these authors contributed equally to this work

ABSTRACT

A second wave pandemic constitutes an imminent threat to society, with a potentially immense toll in terms of human lives and a devastating economic impact. We employ the *epidemic Renormalisation Group* (eRG) approach to pandemics, together with the first wave data for COVID-19, to efficiently simulate the dynamics of disease transmission and spreading across different European countries. The framework allows us to model, not only inter and extra European border control effects, but also the impact of social distancing for each country. We perform statistical analyses averaging on different level of human interaction across Europe and with the rest of the world. Our results are neatly summarised as an animation reporting the time evolution of the first and second waves of the European COVID-19 pandemic. Our temporal playbook of the second wave pandemic can be used by governments, financial markets, the industries and individual citizens, to efficiently time, prepare and implement local and global measures.

Supplementary material

In the Figure we provide plots showing the calibration we used for the simulation case e). The countries represented correspond to those showing early signs of a second wave as of the 5th of August (in addition to Croatia, shown in Fig.2 of the main text).

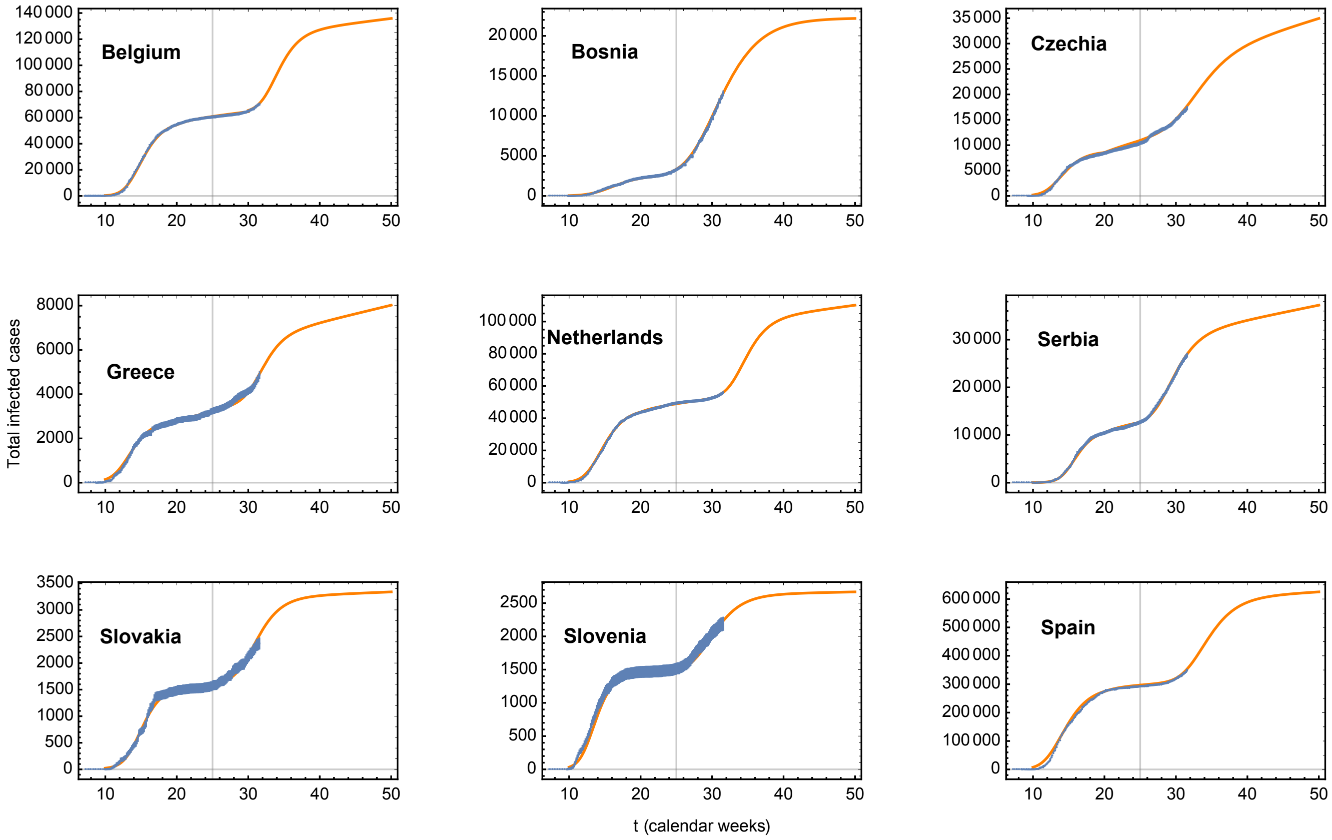

Figure 1. Calibration plots. Number of total infected cases (updated to the 5th of August) with respect to the theoretical curve (orange line) used to calibrate the case e) simulation. The number of cases refer to the total population of each country. The vertical line shows where the second wave simulation begins.
